# Supplementary material for: Tail flaring during agonism in hummingbirds
Source: J Exp Biol. 2026 Oct 7;229(19):jeb253064. doi: 10.1242/jeb.253064 (PMC13723391; doi:10.1242/jeb.253064)
Supplement: Supplementary file 1 [file jexbio-229-253064-s1.pdf]

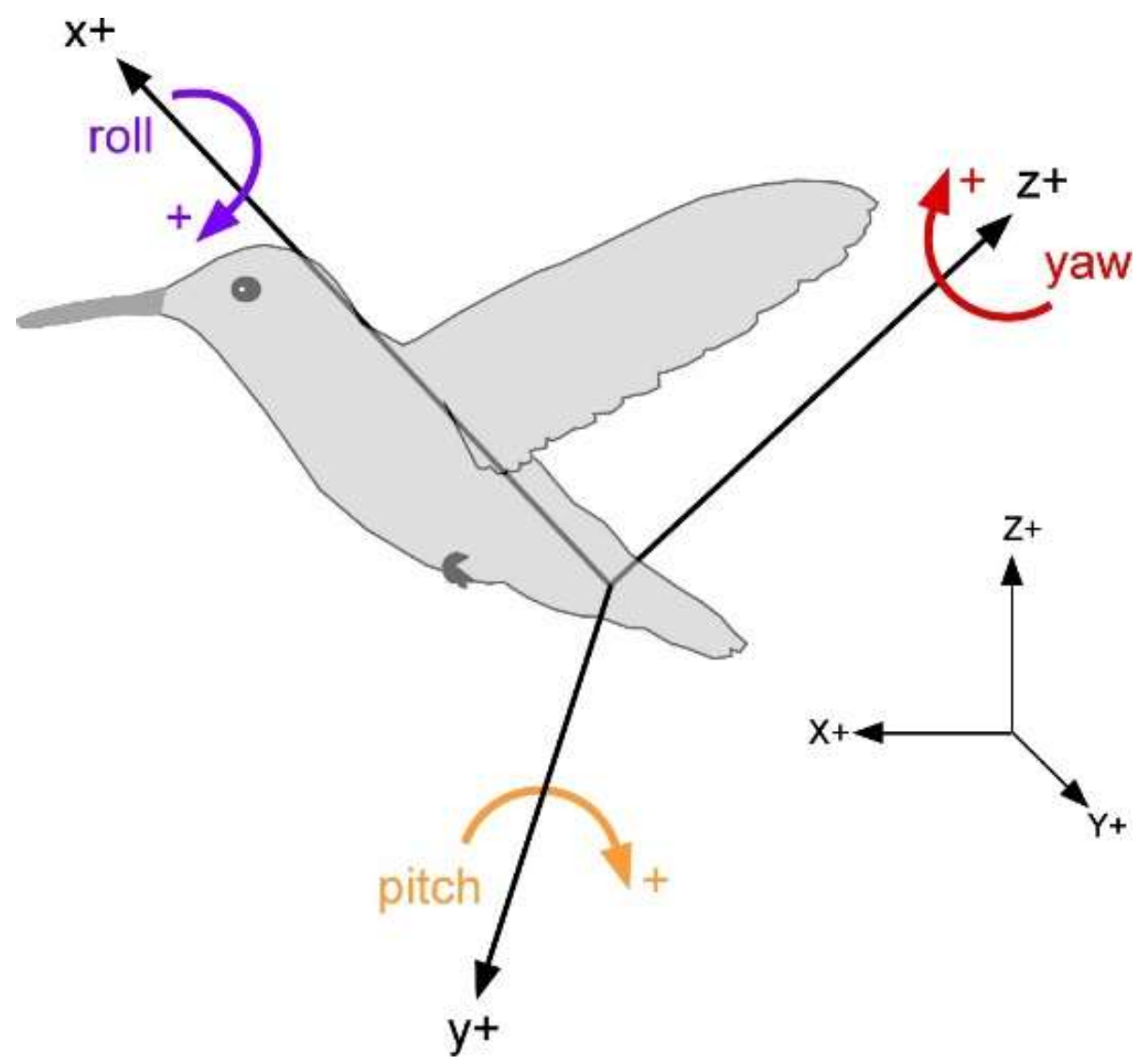

Fig. S1. Global (X, Y, Z) and bird-centered (x, y, z) coordinate systems.

Table S1. Coefficients of variance (CV) in length (mm) of the bill and width of the rump digitized from male Calliope hummingbirds (*Selasphorus calliope*). We include each combination of behavior and individual in our experiments in the competition arena. Rump widths changed as the hummingbirds flared their tails, therefore, we report the Pearson correlation coefficient between rump width and tail angle. In our methods of quality control, we ensured CV < 5% for fixed length of a given bird's bill.

**Bird number**	**Treatment & Outcome**	**Bill CV**	**Rump CV**	**Pearson Correlation Coefficient**
1	Competition (Win)	2.06	27.34	0.90
1	Competition (Loss)	3.61	14.04	0.46
1	Landing	4.35	6.39	-0.43
1	Takeoff	3.55	6.00	-0.41

Journal of Experimental Biology • Supplementary information

2	Competition (Loss)	2.25	13.13	0.48
2	Competition (Win)	2.04	15.73	0.96
2	Landing	2.51	6.03	-0.47
2	Takeoff	4.07	7.50	0.76
3	Competition (Loss)	2.65	10.80	0.79
3	Competition (Win)	2.40	13.12	0.85
3	Takeoff	4.01	8.08	-0.31
3	Landing	3.79	4.92	-0.52
4	Competition (Loss)	3.35	14.62	0.83
4	Competition (Win)	2.18	10.95	0.30
4	Landing	3.89	10.31	-0.83
4	Takeoff	3.98	18.58	-0.55
5	Competition (Loss)	2.37	8.07	0.02
5	Competition (Win)	1.78	14.66	0.69
5	Takeoff	3.91	11.46	-0.51
5	Landing	4.43	15.42	-0.74
6	Competition (Loss)	1.6613	10.9434	0.80
6	Competition (Win)	4.3344	7.8316	-0.05
6	Takeoff	4.5824	9.4907	-0.77
6	Landing	3.0602	6.0064	-0.03
7	Competition (Loss)	3.3796	15.17	0.72
7	Competition (Win)	2.1124	9.0426	0.82
7	Takeoff	1.8674	7.5372	-0.08
7	Landing	2.6583	2.7069	-0.02
8	Competition (Loss)	4.7884	11.4611	0.74
8	Competition (Win)	2.3298	17.137	0.83
8	Takeoff	2.0092	5.2354	-0.36
8	Landing	2.4427	6.6023	-0.68
9	Competition (Loss)	2.5579	9.9052	0.80
9	Competition (Win)	2.9845	18.1459	0.80
9	Takeoff	1.9726	8.5034	-0.076
9	Landing	1.8754	7.0543	-0.81
10	Competition (Loss)	2.5836	5.5003	0.36
10	Competition (Win)	3.9619	12.9912	0.73
10	Takeoff	3.5523	7.2384	-0.69
10	Landing	1.8598	6.2767	-0.59

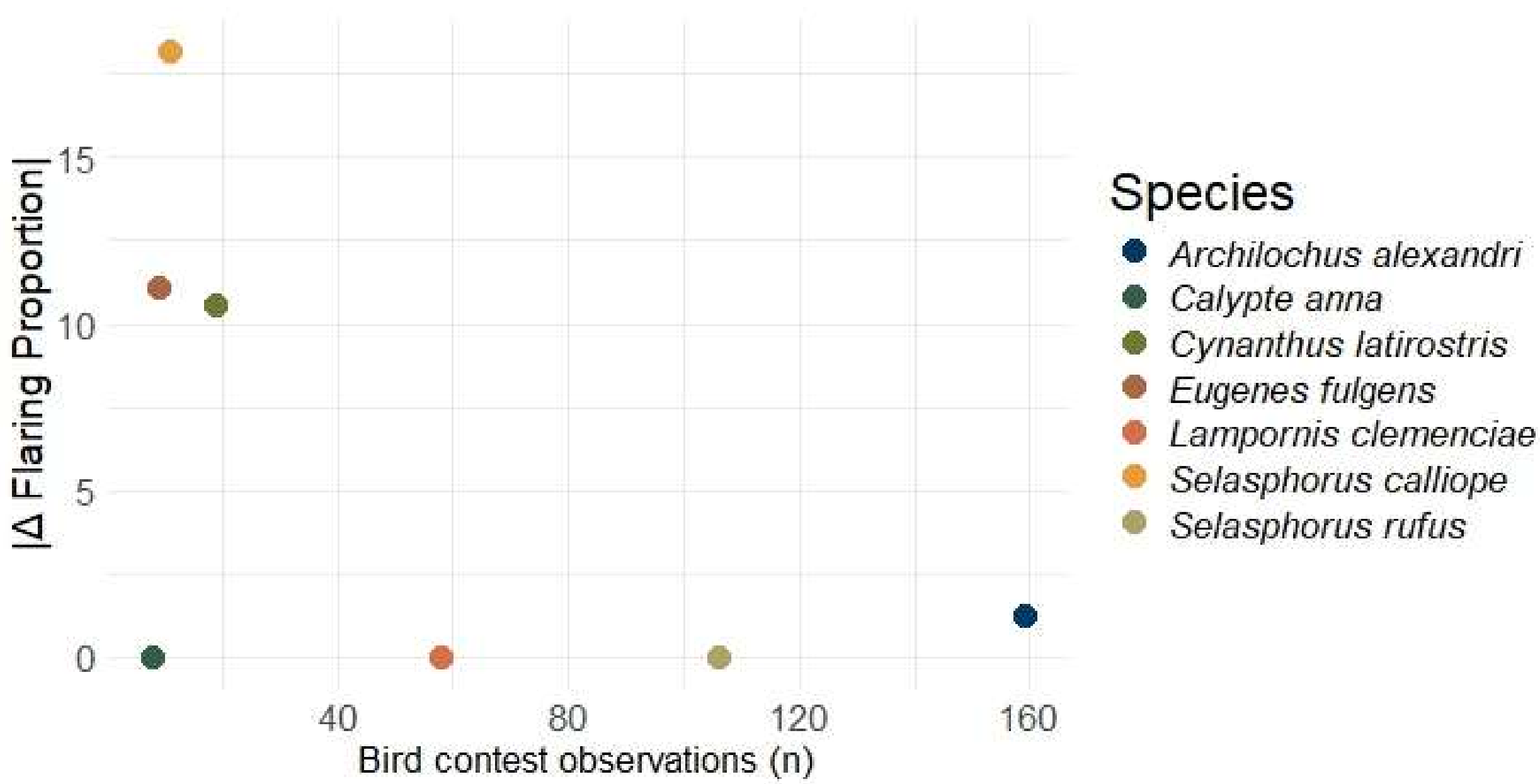

Fig. S2. Graphical summary of sensitivity analysis presented in Table 1. X-axis is total number of bird-contest observations for each species. Y-axis is the absolute change in proportion between the researcher-informed values and cautious values for tail flaring during agonistic encounters in seven species hummingbirds during field study in Arizona, USA.

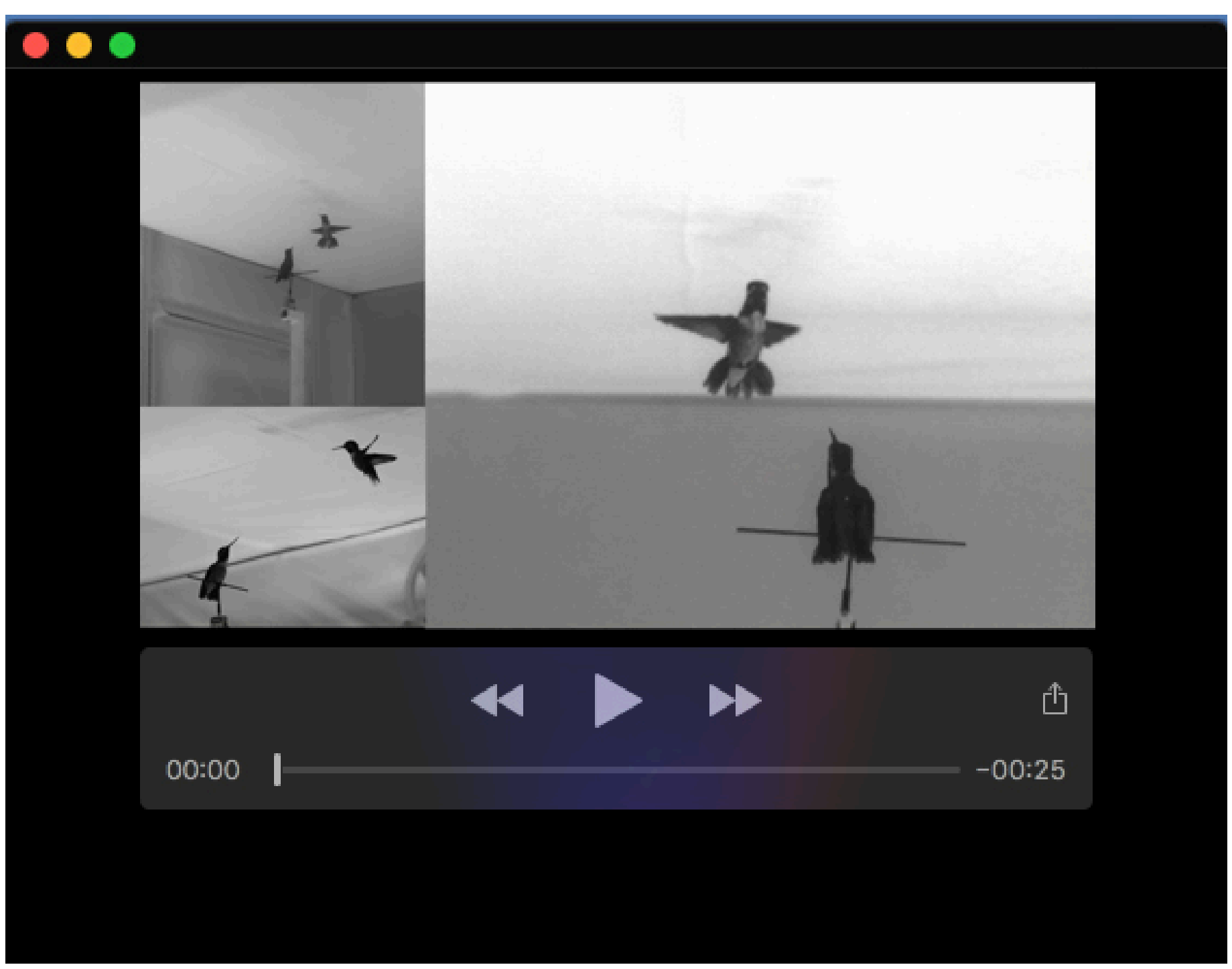

Movie 1. Multiple views of tail flaring by a male Calliope hummingbird (*Selasphorus calliope*) displaying to a male on the high perch (desired resource) in our contest arena. The displaying male (Bird 3 in the main manuscript) regularly displaced competitors and secured the high perch in competitions. Original video sampled at 2000 frames s^{-1}, with playback at 200 frames s^{-1}.

Journal of Experimental Biology • Supplementary information
